# Acute Lung Injury in aortic dissection : new insights in anesthetic management strategies

**DOI:** 10.1186/s13019-023-02223-3

**Published:** 2023-04-17

**Authors:** Ming Yang

**Affiliations:** Department of Anesthesiology, Xinqiao Hospital of Chongqing, Second Affiliated Hospital of Army Medical University, PLA, Chongqing, 400037 China

**Keywords:** Acute aortic dissection, Acute lung injury, Ventilation, Inflammation, Non-coding RNA, Anesthetic management

## Abstract

Acute aortic dissection (AAD) is a severe cardiovascular disease characterized by rapid progress and a high mortality rate. The incidence of acute aortic dissection is approximately 5 to 30 per 1 million people worldwide. In clinical practice, about 35% of AAD patients are complicated with acute lung injury (ALI). AAD complicated with ALI can seriously affect patients’ prognosis and even increase mortality. However, the pathogenesis of AAD combined with ALI remains largely unknown. Given the public health burden of AAD combined with ALI, we reviewed the anesthetic management advances and highlighted potential areas for clinical practice.

## Introduction

Acute aortic dissection (AAD) is a severe cardiovascular disease characterized by rapid progress and a high mortality rate. It seriously endangers the safety of people. The incidence of AAD is approximately 5 to 30 per 1 million people annually worldwide [1]. In emergency departments (ED) in Berlin, acute type A aortic dissection (ATAAD) incidence was 5.24 per 100,000 people. Considering out-of- hospital deaths, the estimated ATAAD incidence was 11.9/100,000 [2, 3]. With the medical advances, the mortality rate has decreased to 0.5% per hour for the first 48 h (23.7% at 48 h) since the onset of symptoms in patients with type A acute aortic dissection with medical treatment [4]. A study has suggested that approximately 35% of AAD patients are diagnosed with concurrent acute lung injury (ALI) [5]. AAD-complicated ALI can result in adverse outcomes, deteriorating the prognosis of patients, and even increasing mortality. Nevertheless, its definite pathogenesis remains unclear. Few data exist on the perioperative anesthetic management of adult patients with AAD combined with ALI. Herein, we focused on the developments of anesthetic management of AAD patients combined with ALI.

## Pathophysiology and epidemiology

Several classification systems have been established for AAD. Among them, Stanford and DeBakey are the most used. Aortic dissection can be divided into Stanford type A, involving the ascending aorta, and Stanford type B, confined to the descending aorta. DeBakey type I is defined as the entire aorta affected; DeBakey type II confines the ascending segment; DeBakey type III confines the descending segment. Acute aortic dissection type A is a highly lethal acute aortic syndrome, with in-hospital mortality of 15–38% [6]. In most cases, AAD pathology starts with intimal injury, leading to false lumen formation. Then, an intimal-medial flap separates the false lumen from the true lumen [7, 8]. Blood malperfusion triggers the propagation of aortic dissection, resulting in the ischemia of involved organs. Meanwhile, an excessive inflammatory response occurs, contributing to the development of oxygen impairment [9]. In Chinese adults patients undergoing ATAAD surgery, 78.5% of them who developed postoperative hypoxemia had a preoperative oxygenation impairment, which might further aggravate the prognosis [10]. However, the pathogenesis of AAD combined with ALI remains largely unknown.

## Cellular and biochemical regulatory pathways

A recent study suggested that inflammation and coagulation are involved in AAD combined ALI [7, 10]. Endothelial and epithelial barriers are destroyed by increased alveolar-capillary barrier permeability, which is responsible for ALI [11]. Furthermore, inflammatory and oxidative stress-related cellular and metabolic regulatory mechanisms might participate in the AAD course worsened by ALI [12, 13]. Herein, we summarized significant mechanisms involved in the progression of AAD combined with ALI.

## NFκB pathway

Nuclear factor kappa-light-chain-enhancer of activated B cells ( NF-κB) can regulate the transcription of several encoding proinflammation cytokines genes and accelerates lung dysfunction [14]. NF-κB signal pathways play a crucial role in the inflammatory response. Angiotensin-II (Ang-II) can be used to establish an animal model of AAD-complicated ALI by stimulating human pulmonary microvascular endothelial cells (PMVEC) [15]. Moreover, Ang-II can participate in the early stages of AAD complex ALI by facilitating the release of matrix metalloproteinases via NF-κB pathway downregulation [15, 16]. Ang-II-induced macrophage infiltration is also closly related to ALI at the onset of AAD [16]. Another in vitro experiment also revealed that Bindarit (Bnd), an indazole derivative, could attenuate the incidence of AAD-complicated ALI by modulating the NF-κB pathway [17].

## JAK2/STAT3 pathway

Interleukin-22 (IL-22) is a protective inflammation factor that plays a critical role in the pathogenesis of autoimmune diseases and anti-apoptosis. Moreover, IL-22 is a member of the IL-10 family, which has protective effects on animal models with ischemia-reperfusion injury in the lung [18]. The JAK/STAT signal pathway is involved in various biological processes [19]. IL-22 can inhibit the apoptosis of PMVECs induced by Ang-II by activating the JAK2/STAT3 pathway [20]. It also has been reported that microRNA-101 can inhibit the proliferation of PMVECs by targeting the JAK2/STAT3 pathway [21].

## HMGB1/RAGE pathway

High-mobility group box 1 (HMGB1) is a nuclear nonhistone protein crucial in initiating and maintaining inflammation [22]. The receptor for advanced glycation end products (RAGE) binds with HMGB1 and activates inflammatory factors such as NF-κB and IL-6 [23]. In AAD complicated ALI, the excessive inflammatory cascade plays a critical role, leading to damaged PMVECs and increased microvascular permeability [24]. HMGB1/RAGE signal pathway exists in various respiratory system diseases, and it might participate in the pathogenesis of AAD-complicated ALI, providing a potential therapeutic target [9].

## Predisposition

Among AAD patients, the most dangerous risk is hypertension. Almost 75% of patients have a history of hypertension, especially poorly controlled. Other risk factors include trauma, drugs, smoking history, and genetic disorders (e.g., Marfan, Loeys-Dietz and Ehlers-Danlos syndromes) [25]. According to a study from the Berlin-Brandenburg region, hypertension, preexisting aortic dilatation, and hereditary connective tissue disease accounted for 62.7, 10 and 1.8%, respectively [3]. A multi-center case-crosser study showed that daily mean temperature lower than 24 °C and temperature drops between neighboring days are associated with increased AAD risk [26].

Obese patients are more predisposed to develop ATAAD complicated ALI [27]. Obesity can trigger chronic inflammation by activating inflammasomes in adipose tissues, promoting the production of cytokines IL-1β and IL- 18 [28]. Obese people are also predisposed to oxygen deficits, resulting in persistent hypoxia in adipose tissue [29]. Moreover, obesity is linked to lung damage due to poor lung compliance and irregular ventilation perfusion. Obesity is also one of the most significant risk factors for developing ALI[27, 29].

Furthermore, the interaction between genetics and the nongenetic-environment is involved in the progression of ALI [30]. Recently, the prevalence of coronavirus disease 19 (COVID-19) has challenged the healthcare system. COVID-19 can bind to the lung pneumocyte type II cells, triggering hyper-inflammation and oxidative stress, subsequently inducing ALI development of [31]. Moreover, a detailed analysis showed that metabolic disturbances caused by type II diabetes mellitus (T2DM) might accelerate ALI incidence through platelet activation, coagulation disorders, endothelial dysfunction, and insulin resistance [31, 32]. Recently, a retrospective study suggested that CPB duration ≥ 257.5 min, left atrium diameter ≥ 35.5 mm, hemoglobin ≤ 139.5 g/l, LVPWT (left ventricular posterior wall thickness) ≥ 10.5 mm, NEUT (neutrophilic granulocyte percentage) ≥ 0.824 might also increase the risk of ALI after ATAAD surgery [33].

## Diagnose

It is crucial for AAD patients to quickly confirm the diagnosis, clarify the classification, and locate the intimal injury site. ATAAD can be misdiagnosed as myocardial infarction, acute coronary syndrome, or cardiac arrest of unknown reasons in emergency department [3]. It is essential to separate the diagnosis of acute aortic dissection from other diseases since treatment differences might lead to disastrous consequences [11]. Among misdiagnosed patients, 81% were determined to have type A aortic dissection [34]. Those walking into the hospital or providing an obscure history might meet increased risks of misdiagnosis[35]. Up to 80% of patients who diagnosed with AAD had received an emergency diagnosis previously [36]. According to a retrospective study, the initial misdiagnosis of AAD was 31% in emergency department [36]. The primary diagnostic imaging method currently used is contrast-enhanced CT angiography. Magnetic resonance imaging (MRI) is well-suited for diagnosing aortic diseases [11]. However, it is limited due to longer acquisition times than CT and the difficulty in long-term monitoring for patients with unstable circulation [37]. In patients with ATAAD complicated ALI, comprehensive evaluation and early diagnosis of ALI have predictive value. According to Berlin’s gold standard definition, the clinical definition of acute respiratory distress syndrome (ARDS) mainly relies on the acute onset, chest imaging of bilateral opacities, and PaO_2_/FiO_2_ ≤ 200 mmHg with positive end-expiratory pressure or continuous positive airway pressure ≥ 5 cmH_2_O; ALI was introduced using the same criteria but with less-severe hypoxemia (i.e., PaO_2_/FiO_2_ ≤ 300 mmHg) [38]. Additionally, to diagnose AAD complicated with ALI, we should exclude pulmonary edema caused by cardiac failure, fluid overload, or other extrapulmonary factors. The degree of bilateral lung opacities is used in the radiographic assessment of lung edema (RALE) score [39]. The score is obtained by summing the chest X-ray analysis score according to density and consolidation. When the RALE score is ≥ 16, 36.3% of patients develop dyspnea and hypoxemia, defined as ALI [40]. In recent years, lung ultrasound has gained popularity for its portability and real-time and noninvasive characteristics. A lung ultrasound score (LUS) has been gradually applied in predicting the severity and prognosis of AAD-complicated ALI. Lung ultrasound is used to evaluate the lung areas, including bilateral, lateral, upper, and lower posterior chest walls, to provide a point score based on the severity of lung tissues: lung consolidation = 3 points, severely reduced lung ventilation area = 2 points; moderately reduced lung ventilation area = 1 point; normal ventilation area = 0 points. The cutoff values for the LUS score in ALI patients are 18.6 points [41, 42]. As PaO2/FiO2 changes occur after the lung ventilation area change, the LUS can well reflect lung changes and evaluate ALI severity.

### Aortic dissection detection risk score

A pre-test probability assessment of the risk scores of aortic dissection detection (ADD-RS) might improve AAD diagnosis and decrease the misdiagnosis rate [43–45]. ADD-RS can be used in preclinical settings to optimize diagnostics. The major components of risk assessment tools include relevant history, pain features, and clinical examination findings [44], and high scores indicate a stronger AAD suspicion. For example, A multicenter prospective study including 1850 patients showed that AAD-RS ≤ 1 with D-dimer < 500 ng/mL had a missing rate of 1/300 cases of acute aortic syndromes; for patients with AAD-RS > 1 and D-dimer < 500 ng/mL, the incidence of acute aortic syndrome was 1/22 [43]. Another retrospective study incorporating the ADD-RS system in emergency care found that patients with ATAAD had a shortened mean time from 8.6 to 5.5 h from the initial symptoms to surgery and a decreased initial misdiagnosed rate in patients with AAD-RS ≥ 2 [46].

## Preoperative evaluation and management

Considering the high mortality rate, a study suggested that the time from symptom onset to surgery in patients with ATAAD should not exceed 10 h [6]. When combined with ALI, preoperative oxygen impairment might occur, increasing the incidence of postoperative acute respiratory distress syndrome [17]. Therefore, surgeons and anesthesiologists must schedule a detailed preoperative evaluation. Considering postoperative pulmonary complications, there is still a debate on surgery time. In our center, we choose early operation because AAD can aggravate the inflammatory cascade.

For patients with ATAAD, initial management aims to control blood pressure and pain, which can limit the false lumen propagation [47]. Blood pressure control can lower shear strain on the arterial wall and increase organ tissue perfusion. The recommended systolic blood pressure is 100–120 or 70–90 mmHg for typical arterial blood pressure, with a heart rate of 60–80 beats per minute [25]. Beta-blocker is recommended as the first-line drug that can simultaneously reduce blood pressure and heart rate to minimize aortic shear stress [6, 48]. Among them, propranolol, metoprolol, labetolol, and esmolol are generally preferred to control initial blood pressure, and calcium channel blockers such as verapamil or diltiazem, are more suitable for asthma or bradycardia patients [49]. Additionally, recent reports have shown that Beta-blocker has an anti-inflammatory effect, which can alleviate lung oxygen impairment in ATAAD-complicated ALI [48]. Other studies have shown that beta-agonist can accelerate the resolution of pulmonary edema by regulating type I and type II cells but increase the mortality rate [38]. Opiate is currently underway for pain management in AAD in clinical practice to reduce the sympathetic release of catecholamines [50].

Both inflammation and coagulation are involved in ATAAD-complicated ALI. Coagulation disorder is usually associated with low coagulation factors and high D-Dimer in ATAAD patients. Moreover, secondary fibrinolysis caused by ATAAD can worsen ALI [10]. Therefore, better control of abnormal coagulation is essential. Preoperative hypoxemia might occur in most ATAAD-complicated ALI patients. The optimal oxygenation target in patients with ALI patients is unclear. However, oxygen toxicity has attracted increasing attention. A randomized clinical trial has suggested that conservative oxygen therapy, maintaining PaO2 between 70 and 100 mmhg or arterial oxyhemoglobin saturation between 94% and 98%, can improve the prognosis [51]. For patients with PaO_2_/FiO_2_ < 200 mmhg, keeping PaO_2_ 55–80 mmHg or SpO_2_ 88-95% and pH ≥ 7.25 might lead to improved outcomes [38].

Assessment of pulmonary risk is essential for AAD-complicated ALI patients. A series of predicting risk indexes for evaluating postoperative respiratory failure have been made based on emergency surgery, a history of smoking, and chronic obstructive pulmonary disease [52]. Arterial blood analysis can predict pre-anesthesia hypoxemia and provide timely treatment. Point of Care Ultrasound (POCUS) of the lung is also indicated to evaluate preoperative pulmonary disease, which can be used as clinical treatment. Pulmonary ultrasound can be implemented bedside, real-time, presenting substantial advantages in diagnosing ALI and providing references for clinicians to make decisions [42].

## Intraoperative management

### Monitoring

Swift surgical treatment is essential to repair AAD. Complex monitoring is currently suggested in clinical practice, including invasive arterial blood pressure, central venous pressure, nervous system, depth of anesthesia, and regional cerebral oxygen saturation. Transesophageal echocardiography has an advantage in diagnosing acute intraoperative hemodynamic instability. Therefore, it can be used to evaluate aortic dissection-related cardiac complications, which can lead to, or aggravate, acute respiratory distress syndromes, such as aortic regurgitation, proximal coronary artery involvement, presence of pleural effusion, and compression of pulmonary artery [53]. TEE can also image lung and pleural diseases in critical illness patients, providing an estimate of pulmonary vascular and flow velocity. Pulmonary Artery catheter (PAC) can provide unique hemodynamic data. Thus, it can be used in critical patients, especially in high-risk surgery, heart failure, and acute respiratory dissection syndrome patients. However, early use of PAC cannot affect mortality and organ functions [54, 55]. Moreover, we should prevent the incidence of arrhythmias, catheter knotting, and even pulmonary artery rupture when placing a catheter [56].

### Induction of anesthesia

The primary principle of induction is sustaining a stable circulation. Perioperative aspiration prevention is also important during emergency cardiac surgery. Gastric ultrasound accurately identifies and measures gastric volume and contents, qualifying a safe airway assessment and minimizing aspiration risk [57]. For hemodynamically unstable patients, invasive blood pressure should be performed before anesthesia to identify rapid hemodynamic changes. A defibrillator pad should be pre-applied for those with arrhythmia in case of malignant arrhythmia [58]. Hypovolemia and vasodilation during the induction of anesthesia should also be avoided.

### Choice of anesthetics

Volatile and injectable anesthetics can be employed in cardiovascular surgery. For patients with stable hemodynamics, totally intravenous anesthesia(TIVA) has fewer side effects such as nausea and vomiting, and a lower risk of developing malignant hyperthermia. Pretreatment with volatile agents has cell-protective effects through intracellular signaling pathways, gene expression, and changes in mitochondrial function in the animal models, and it protects against ischemia-reperfusion injury. Volatile agents can also reduce myocardial infarction [59]. However, a prospective analysis of 5400 individuals undergoing elective cardiac surgery found no significant difference in mortality after anesthesia with a volatile agent or completely intravenous anesthetics after one year [60]. However, volatile agents are associated with shorter duration of hospital and ICU stays [61]. Volatile anesthetics have a lung-protective effect on ALI [62]. Therefore, it is more suitable for AAD-complicated ALI patients. Besides, sevoflurane during cardiopulmonary bypass might affect inflammatory response [63]. Pretreatment with sevoflurane can attenuate lung injury by inhibiting neutrophil accumulation and attenuating the inflammatory response. Exposure to isoflurane can also attenuate neutrophil recruitment to the lungs in an animal model with abdominal sepsis [64]. Therefore, inhaled anesthetics might be a good choice for AAD-complicated ALI patients.

### Fluid management

Perioperative lung injury is a common complication in patients undergoing Sun’s surgery. Thus, conservative fluid management is essential. Fluid overload may aggravate lung injury, increasing ICU stay and mortality risk. Gelatin and other colloids have been used in heart surgery to enhance plasma volume. However, their effects on renal function are debatable. A recent report showed that 4% succinyl gelatin administration was not associated with an increased risk of kidney injury in cardiac surgery compared to the crystalloid-only group [65]. Fluid management surgeries significantly impact the clinical outcomes of AAD-complicated ALI patients. A major randomized controlled trial with ICU patients found that a conservative hydration regimen was linked to fewer ventilating days and better oxygenation [66]. Early fluid overload might further increase the mortality of ALI patients [67]. Transfusion-related acute lung injury (TRALI) is characterized by pulmonary permeability edema. TRALI is defined as acute onset hypoxemia, and bilateral pulmonary infiltrates during or within 6 h of blood product transfusion. Among patients undergoing cardiac surgery, approximately 2.4% might develop TRALI [68]. A retrospective study showed that intraoperative packed red blood cell (pRBC) and fresh frozen plasma (FFP) transfusion were related to postoperative mechanical ventilation time in ATAAD patients undergoing total aortic arch replacement [69]. Recently, the principle of goal-directed fluid therapy (GDFT) guided by hemodynamic measures has been shown to reduce complications after various surgeries. Nevertheless, more investigations on GDFT in AAD surgery are warranted.

### Ventilation management

For preoperative ALI, we suggest a low-tidal volume protective ventilation mode (6–8 mL/kg predicted body weight) to reduce the incidence of ventilator-induced lung injury. Permissive hypercapnia is a lung protective ventilation strategy widely used in recent years. A recent prospective investigation in elective cardiac surgery patients demonstrated that maintaining the PaCO2 at 46–60 mmhg could increase cerebral blood flow and improve the cerebral oxygen supply/consumption balance [70]. Cardiac surgery with cardiopulmonary bypass induces abnormal inflammatory responses, accelerating lung injury.

Mechanical ventilation remains the mainstay management for AAD- complicated ALI patients. However, it can bring potential injuries to ALI patients. The underlying mechanism of lung injury caused by ventilation can be divided into volutrauma and aelectrauma, which can aggravate the leakage of plasma-derived fluid and proteins into the mesenchyme of the lung [71]. Thus, an intraoperative ventilation strategy to maintain adequate gas exchange while protecting lungs from inflammation can improve postoperative pulmonary complications. In recent years, driving pressure and mechanical power are commonly used in reducing ventilator-induced lung injury.

Driving pressure is defined as plateau pressure (Pplat) minus PEEP, representing the stress and strain on the lung. For patients undergoing general anesthesia, intraoperative high driving pressure is associated with more postoperative pulmonary complications [72]. A prospective study including 122 acute respiratory distress syndrome patients found that the best PEEP level by driving pressure (10 ± 4.1 cmH_2_0) was lower than the best PEEP by PaO_2_/FiO_2_ (11.9 ± 4.7 cmH20). They also suggested that the best method for PEEP should affect patient outcomes [73]. A recent randomized controlled study found that using a driving pressure-guided breathing method during cardiac surgery can reduce the risk of postoperative pulmonary complications (PPCs). They used protective ventilation strategies with total volume (V_T_) < 8 mL/kg ideal body weight, modified driving pressure (peak inspiratory pressure - PEEP) < 16 cmH_2_O, and PEEP ≥ 5 cmH_2_O, and demonstrated that it is the driving pressure associated with the lower likelihood of PPCs [74].

Mechanical power is the energy per unit time generated by ventilation applied to respiratory system [75]. A retrospective study with 8207 critical patients who needed ventilation in the ICU showed that a mechanical power higher than 17 J/min was associated with higher hospital mortality [76]. However, the available threshold of mechanical power for developing ventilator-induced lung injury in AAD with ALI remains unclear.

Intraoperative lung recruitment has been proposed since the 1960s. It is defined as an inspiratory hold with 40 cmH2O of inspiratory pressure for 20–30 s to prevent atelectasis. However, we should be cautious about its effects on the left ventricular end-diastolic and cardiac output. A recent study on patients undergoing on-pump cardiac surgery proved that intensive alveolar recruitment procedures (continuous positive airway pressure maintained at 30 cmH2O for 30 s) would be more suitable for patients with hypoxemia or significant lung atelectasis rather than a preventive approach [77]. They also demonstrated that maintaining ventilation during CPB combined with perioperative recruitment maneuvers does not improve the incidence of postoperative pulmonary complications compared to no ventilation. Moreover, atelectasis brought by lung maneuver would recur within 40 min [78].

The debate on ventilation during CPB is still going on. An argument states that it can improve gas exchange and oxygen index. A prospective randomized trial showed that maintaining ventilation during CPB cannot reduce PPCs in cardiac surgery patients [79]. Another multicenter research found that open lung ventilation combined with CPB breathing does not retain the advantages after surgery, which might be related to a higher number of inflammatory factors [80]. Considering the fraction of inspired oxygen (FiO_2_) management, a high oxygen concentration might lead to absorption atelectasis and even aggravate lung injury. Besides, moderate hyperoxia (50–80% FiO_2_) might reduce the incidence of surgical infections. However, more studies are required to determine the optimum FiO_2_ to prevent infections and atelectasis in cardiac surgeries [81]. Athough the existing studies have given many strategies for ventilation, the small sample size limited clinical use.

### Postoperative management

An optimal postoperative management schedule might benefit preoperative ALI patients. For AAD-complicated ALI patients, postoperative pulmonary complications after cardiac surgery can increase the mortality in the ICU. A more intensive alveolar recruitment strategy (patients receiving three cycles of lung inflation, 60 s each, PEEP of 30 cmH_2_O, driving pressure of 15 cmH_2_O, respiratory rate of 15/min, inspiratory time of 1.5 s, and FiO_2_ of 40%) in the postoperative phase is associated with fewer PPCs, shorter ICU lengths of stay, lower use of supplement oxygen, and extended noninvasive mechanical ventilation [82]. Furthermore, Andreas and his colleges designed a postoperative recruitment maneuvers protocol in patients undergoing uncomplicated cardiac surgery with stable hemodynamics :first step: increase the PEEP from 5 to 20 cmH_2_O over 30 s; second step: change the ventilator mode to pressure control with an inspiratory pressure of 20 cmH_2_O for 30 s; third step: decrease the PEEP to 10 cmH_2_O over 2 min [83]. They found that the recruitment maneuvers protocol in a prone position could improve lung aeration and oxygenation.

PEEP titration can reverse atelectasis. Borges scheduled a PEEP titration way to choose an optimal PEEP level: started with 25 cmH_2_O PEEP, then decreased 2 cmH_2_O and maintained at this level for 4 min, until the PaO_2_ + PaCO_2_ < 380 mmHg. The optimum PEEP was the lowest PEEP maintaining the sum of blood gases ≥ 400 mmHg [84]. Through multislice computed tomography (CT) and continuous blood gas, they found that the maximum-recruitment strategy combined with the PEEP titration could reverse hypoxemia and fully recruit the lung in early ARDS. Another multi-center study compared a schedule with the same lung recruitment and titrated PEEP strategy with low PEEP ventilation in moderate to severe ARDS patients [85]. They showed an increased 28-day mortality rate in the PEEP titrating group than in the low PEEP group. Moreover, the lung recruitment strategy increase the 6-month mortality and hypotension incidence in the first hour. Therefore, they do not suggest a commonly use of lung recruitment and titrated PEEP in these patients. AAD-complicated ALI patients often show rough hemodynamics, and it is more difficult to carry out an optimal titrated PEEP schedule.

Point-of-care lung ultrasound can guide ventilation strategy and assess ALI severity during the postoperative period. Pulmonary ultrasound has more sensitivity than X-ray for detecting lung abnormality. A prospective study enrolling 109 patients undergoing major surgery admitted to the intensive care unit found that lung ultrasound abnormalities were associated with postoperative pulmonary complications, and ultrasound would promote earlier intervention and improve the prognosis [86]. Moreover, lung ultrasound has gained widespread use in the ICU for assessing pulmonary disease, providing a reliable diagnosis.

## Treatment

ALI management remains conservative and lacks established target therapies. Currently, treatments for improving the pathophysiology of ALI have attracted significant attention. Herein, we discuss drug and non-drug therapies.

## Drug therapy

Since inflammation plays an important role in AAD-complicated ALI, we first discuss anti-inflammatory treatment. Nitric oxide (NO) is a vasodilator, especially for pulmonary hypertension patients. It can enhance arterial oxygen, regulate inflammatory-induced microvascular injury in the lung, and decrease the need for ventilation. A recent study showed that NO could reduce pulmonary and systemic neutrophils and pre-inflammatory factors in lung injury in a rodent ARDS model [87]. Dexmedetomidine (DEX) is a selective α2-adrenergic receptor agonist clinically used for sedation and analgesia. DEX has antioxidant, anti-apoptosis, and anti-inflammatory properties [88]. Studies have shown that DEX can activate the protein kinase C/heme oxygenase-1 pathway and decrease LPS-induced ALI [89]. In a prospective clinical trial, Xenon, an ideal anesthetic with hemodynamic stability and anti-inflammatory effects, eased lung injury in cardiac surgery [90]. In their study, pulmonary static inflation with 50% Xenon during CPB can triggered anti-inflammatory responses and suppressed pro-inflammatory and oxidative effects. In another sepsis-induced ALI model, the antioxidant idebenone (IDE) alleviated inflammatory response by reducing free radicals and lipid peroxidation [91]. Escitalopram is clinically used as an antidepressant drug and can inhibit lung inflammation through the SIK2/HDAC4/NF-kB pathway [92]. Molecular hydrogen is the lightest chemical element. Its application for ALI is mainly based on anti-inflammatory, antioxidative, autophagy, and cell death effects [93]. Pterostilbene (PTE) is a polyphenol. Pretreatment with PTE can reduce the inflammatory response in an LPS-induced ALI rat model by activating nuclear receptor subfamily 4 group A member (NR4A1) [94]. However, more prospective studies are encouraged to prove their effectiveness in clinical practice.

### Neuromuscular blockade

The use of neuromuscular blocking agents (NMBA) in ARDS adults is debatable. In ARDS patients undergoing mechanical ventilation, NMBA might improve oxygenation and decrease mortality. A multicenter prospective trial showed that using NMBA in early severe ARDS with low tidal volume ventilation could improve the adjusted 90-d survival [95]. A recent evidence-based guideline found that NMBA is more suitable for ARDS patients who require deep sedation to facilitate lung protective ventilation. However, they suggested avoiding the routine use of NMBA in adult patients with any severity [96]. Unfortunately, there is no evidence of the use of NMBA in AAD complicated ALI patients.

## Non-drug therapy

Veno-venous extracorporeal membrane oxygenation (VV-ECMO) is another essential tool to treat ALI. VV-ECMO has been increasingly used in ALI patients, especially for those whose conventional ventilation management fails to supply adequate oxygen exchange [97]. Additionally, postoperative ventilation might lead to secondary lung injury. For AAD-complicated ALI, alveolar overdistension and high transpulmonary pressure can result in trauma, which might also aggravate lung injury. VV-ECMO can mitigate lung injury caused by ventilation, even lung-protection ventilation [98]. Optimized ventilation for patients with ECMO remains controversial. For example, an acute respiratory distress syndrome (ARDS) model requiring ECMO was established to investigate a moderate PEEP to reduce lung injury. They suggested that a 10 cmH_2_O PEEP can minimize lung injury, improve gas exchange, and not affect hemodynamic stability [99]. However, it needs more clinical studies to certify its benefits.

Extracorporeal carbon dioxide removal (ECCO_2_R) can improve acidosis via CO2 clearing. ECCO_2_R combined with ultra-lung protective ventilation, driving pressure, titrating PEEP, or optimizing mechanical power might have more lung protective effects. A prospective study found that using ECCO_2_R and ultra-protective ventilation could mitigate respiratory acidosis in moderate ARDS patients [100]. However, contraindications for systemic anticoagulation and bleeding disorders are crucial in cardiac surgery patients. Hence, the overall benefits and harms should be addressed in the future.

## Conclusion

Among patients with preoperative ALI complicated with AAD, early and rapid assessment of ALI and guidance for early management results in lower mortality. Combining the detection risk scores of AAD could decrease the misdiagnosis rate, which should be validated in more prospective studies. Moreover, the recommendations regarding the treatments, and intervention strategies to better manage AAD-complicated ALI patients including ventilation mode, drug therapy, extracorporeal membrane lung need further investigation. Improving diagnostic skills, transfer system implementation and enhancing communication with departments may shorten the time from the onset to surgery in patients with ATAAD. Increased awareness might further aid AAD diagnosis. More randomized clinical trials are required to optimize the perioperative management.

## Data Availability

Not applicable.

## Abbreviations

AAD: Acute aortic dissection
ATAAD: Acute Type A aortic dissection
ALI: Acute lung injury
NF-κB: Nuclear factor kappa-light-chain-enhancer of activated B. cells
Ang-II: Angiotensin-II
PMVEC: Pulmonary microvascular endothelial cells
Bnd: Bindarit
IL-22: Interleukin-22
HMGB1: High-mobility group box 1
COVID-19: Coronavirus disease 19
T2DM: Type II diabetes mellitus
ARDS: Acute respiratory distress syndrome
RALE: Radiographic assessment of lung edema
LUS: Lung ultrasound score
POCUS: Point of Care Ultrasound
TEE: Transesophageal echocardiography
PAC: Pulmonary Artery catheter
TIVA: Totally intravenous anesthesia
TRALI: Transfusion-related acute lung injury
pRBC: Packed red blood cell
FFP: Fresh frozen plasma
GDFT: Goal-directed fluid therapy
Pplat: Plateau pressure
PPCs: Postoperative pulmonary complications
FiO_2_: Fraction of inspired oxygen
NO: Nitric oxide
DEX: Dexmedetomidine
PTE: Pterostilbene
NR4A1: Nuclear receptor subfamily 4 group A member
NMBA: Neuromuscular blocking agents
VV-ECMO: Veno-venous extracorporeal membrane oxygenation
ECCO_2_R: Extracorporeal carbon dioxide removal
MRI: Magnetic resonance imaging
